# Supplementary material for: Toxin/antitoxin systems induce persistence and work in concert with restriction/modification systems to inhibit phage
Source: Microbiol Spectr. 2023 Dec 6;12(1):e03388-23. doi: 10.1128/spectrum.03388-23 (PMC10783111; doi:10.1128/spectrum.03388-23)
Supplement: Supplemental material — Tables S1 to S6 and Fig. S1 to S4. [file spectrum.03388-23-s0001.docx]

**Supporting Information**

**Toxin/Antitoxin Systems Induce Persistence and Work in Concert with Restriction/Modification Systems to Inhibit Phage**

**Laura Fernández-García**^1,2^**, Sooyeon Song**^1,3,4^**, Joy Kirigo**^1^**, Michael E. Battisti**^1^**, Maiken E. Petersen**^1,5^**, María Tomás**^2^**, and Thomas K. Wood**^1*^

^1^Department of Chemical Engineering, Pennsylvania State University,

University Park, Pennsylvania, 16802-4400, USA

^2^Microbiology Translational and Multidisciplinary (MicroTM)-Research Institute Biomedical A Coruña (INIBIC) and Microbiology Department of Hospital A Coruña (CHUAC); University of A Coruña (UDC), A Coruña, Spain

Departments of Animal Science^3^ and Agricultural Convergence Technology^4^, Jeonbuk National University, 587 Baekje-Daero, Deojin-Gu, Jeonju-Si, Jellabuk-Do, 54896, South Korea

^5^Interdisciplinary Nanoscience Center, Aarhus University, 8000 Aarhus C, Denmark

*For correspondence. E-mail [tuw14@psu.edu](mailto:tuw14@psu.edu)

Tel. (+)1 814-863-4811; Fax (1) 814-865-7846

**Table S1. Bacterial strains and plasmids utilized.** Cm^R^ is chloramphenicol resistance, and Kan^R^ is kanamycin resistance.

| **Strains and Plasmids** | **Features** | **Source** |
| --- | --- | --- |
| **Strains** |  |  |
| *E. coli* MG1655 | F^-^ lambda- *ilvG*- *rfb*-50 *rph*-1 | ([1](#_ENREF_1)) |
| *E. coli* BW25113 | *rrnB3 ΔlacZ4787 hsdR514 Δ(araBAD)567 Δ(rhaBAD)568 rph-1* | ([2](#_ENREF_2)) |
| *E. coli* BW25113 *mcrB* | BW25113 *mcrB* Ω Kan^R^ | ([2](#_ENREF_2)) |
| *E. coli* BW25113 *recD* | BW25113 *recD* Ω Kan^R^ | ([2](#_ENREF_2)) |
| *E. coli* BW25113 *mrr* | BW25113 *mrr* Ω Kan^R^ | ([2](#_ENREF_2)) |
|  |  |  |
| **Plasmids** |  |  |
| pCV1 | Cm^R^ | ([3](#_ENREF_3)) |
| pCV1-*mqsRAC* | P_mqsRAC_::*mqsRAC;* Cm^R^ | ([3](#_ENREF_3)) |
|  |  |  |
| **Phages** |  |  |
| T2 |  | ATCC 11303-B2 |

**Table S2. qRT-PCR primers**.

| **Gene** | **Primer pair** | **Length** | **Sequence (5’ to 3’)** | **T_m_ (ºC)** | **Product size** |
| --- | --- | --- | --- | --- | --- |
| ***rrsG***  ***(housekeeping)*** | *rrsG* Fow | 20 bp | TATTGCACAATGGGCGCAAG | 56.9 | 234 bp |
|  | *rrsG* Rev | 20 bp | ACTTAACAAACCGCCTGCGT | 50.0 |  |
| ***raiA*** | *raiA* Fow | 25 bp | CCAGCAAACAAATGGAAATTACTCC | 61.3 | 122 bp |
|  | *raiA* Rev | 22 bp | TTGTGGCTCTTTGGACAGAATG | 60.8 |  |
| ***rmf*** | *rmf* Fow | 24 bp | AACGGGCACATCAACGTGGTTATC | 64.6 | 100 bp |
|  | *rmf* Rev | 20 bp | CCTCCCAGCCATTGTGACCT | 64.5 |  |
| ***hpf*** | *hpf* Fow | 20 bp | TCGAGATCACCGAGGCACTG | 64.5 | 119 bp |
|  | *gpf* Rev | 24 bp | GGTGTGGGTGACTTTCTCCACTTT | 64.6 |  |
| ***hflX*** | *hflX* Fow | 22 bp | TTCCGCCGGTGTCGAAGCATTG | 66.4 | 124 bp |
|  | *hflX* Rev | 20 bp | CGAAGCACCCGTCGCTTTGA | 64.5 |  |

**Table S3. MqsR/MqsA/MqsC inhibits T2 phage by forming persister cells.** Cells were contacted first with T2 phage at 0.1 MOI for 1 h followed by treatment with antibiotics: 10 MIC (100 µg/ml) for ampicillin, 100 MIC for ciprofloxacin (5 µg/ml), and 5 MIC for mitomycin C (10 µg/ml) for 3 h. Bold results indicate million-fold difference for inhibiting T2 phage. One average deviation shown. Survival percentages are normalized based on no phage addition.

| **Strain** | **Elapsed time, h** | **Condition** | **CFU/ml average** | **Survival, %** |
| --- | --- | --- | --- | --- |
| MG1655/pCV1 | 0 | No Phage | 1.5 ± 0.6 x 10^8^ | 100 % |
|  | 1 | Phage T2 | 9 ± 4 x 10^2^ | 0.0008 ± 0.0005 % |
|  | 3 | No phage + ampicillin | 2 ± 2 x 10^5^ | 0.2 ± 0.3 % |
|  | 3 | No Phage T2 + ciprofloxacin | 3 ± 1 x 10^4^ | 0.011 ± 0.005 % |
|  | 4 | Phage T2 + ampicillin | 0 ± 0 | 0 ± 0 % |
|  | 4 | Phage T2 + ciprofloxacin | 0 ± 0 | 0 ± 0 % |
| MG1655/pCV1-*mqsRAC* | 0 | No Phage | 1.7 ± 0.6 x 10^8^ | 100 % |
|  | 1 | Phage T2 | 6 ± 6 x 10^7^ | 30 ± 30 % |
|  | 3 | No phage + ampicillin | 1 ± 1 x 10^5^ | 0.09 ± 0.05 % |
|  | 3 | No phage + ciprofloxacin | 3.8 ± 0.6 x 10^4^ | 0.017 ± 0.007 % |
|  | 3 | No phage + mitomycin C | 8 ± 9 x 10^1^ | 0.000005 ± 0% |
|  | 4 | Phage T2 + ampicillin | 4 ± 2 x 10^3^ | 0.06 ± 0.07 % |
|  | 4 | Phage T2 + ciprofloxacin | 1.0 ± 0.4 x 10^6^ | 2 ± 2 % |
|  | 4 | Phage T2 + mitomycin C | 2 ± 1 x 10^2^ | 0.000034 ± 0% |

**Table S4. Efficiency of plating and efficiency (EOP) of the center of infection assay (ECOI).** (**A**) EOP assay with the *E. coli* strains used in this manuscript. (**B**) ECOI assay with the strains used in this manuscript. The efficiencies are relative to the strain marked ‘host’. One average deviation shown.

**A**.

| Strain | PFU/mL | EOP vs host | EOP vs empty plasmid |
| --- | --- | --- | --- |
| MG1655 (host) | 9 ± 2 x 10^9^ | 1 |  |
| BW25113/pCV1 | 5.2 ± 0.2 x 10^9^ | 6.0 x 10^-1^ | 1 |
| BW25113/pCV1-*mqsRAC* | 1.9 ± 0.3 x 10^6^ | 2.2 x 10^-4^ | 3.7 x 10^-4^ |
| MG1655/pCV1 | 3.7 ± 0.4 x 10^9^ | 4.3 x 10^-1^ | 1 |
| MG1655/pCV1-*mqsRAC* | 1.30 ± 0.07 x 10^6^ | 1.5 x 10^-4^ | 3.5 x 10^-4^ |
| BW25113 Δ*mcrB/*pCV1 | 4.0 ± 0.5 x 10^10^ | 4.6 x 10^-0^ | 1 |
| BW25113 Δ*mcrB/*pCV1-*mqsRAC* | 1.6 ± 3 x 10^8^ | 1.8 x 10^-2^ | 4.0 x 10^-3^ |

**B**.

| Strain | PFU/mL | ECOI vs host | ECOI vs empty plasmid |
| --- | --- | --- | --- |
| MG1655 (host) | 3.7 ± 0.1 x 10^7^ | 1.000 |  |
| BW25113/pCV1 | 1.43 ± 0.07 x 10^7^ | 0.387 | 1.000 |
| BW25113/pCV1-mqsRAC | 2.9 ± 0.5 x 10^6^ | 0.078 | 0.201 |
| MG1655/pCV1 | 2.6 ± 0.4 x 10^7^ | 0.691 | 1.000 |
| MG1655/pCV1-mqsRAC | 1.8 ± 0.8 x 10^6^ | 0.048 | 0.069 |
| BW25113 Δ*mcrB/*pCV1 | 2 ± 1 x 10^8^ | 5.495 | 1.000 |
| BW25113 Δ*mcrB/*pCV1-mqsRAC | 9 ± 7 x 10^6^ | 0.252 | 0.046 |

**Table S5. Percentage of *E. coli* persister cell resuscitation after treatment with T2 phage (0.1 MOI) for 1 h.** Values in bold are included in the main text, and values for the number of cells with the indicated phenotype are listed parenthetically.

| **Sample** | **No of cells** | **% of waking in 0.5 h** | **% of waking in 0.5 to 3 h** | **% Elongated cells** | **% of dead** |
| --- | --- | --- | --- | --- | --- |
| 1 | 33 | - | 88% (29/33) | 9% (3/33) | 3 % (1/33) |
| 2 | 25 | 20% (5/25) | 12% (3/25) | 16% (4/25) | 52 % (13/25) |
| **3** | **27** | **41% (11/27)** | **33% (9/27)** | **11% (3/27)** | **15 % (4/27)** |
| 4 | 66 | 58% (38/66) | 15% (10/66) | 18% (12/66) | 9 % (6/66) |
| 5 | 24 | 25% (6/24) | 21% (5/24) | 33% (8/24) | 21 % (5/24) |
| **Average** |  | 36 ± 13% | 34 ± 22% | 18 ± 7% | 20 ± 13% |

**Table S6. Phage attack does not induce ribosome inactivation proteins.** Fold changes for the wild-type *E. coli* with the empty plasmid pCV1 relative to the wild-type producing MqsR/MqsA/MqsC (from pCV1-*mqsRAC*) after 15 min of T2 attack as determined by qRT-PCR. Cycle numbers (C_t_) are indicated for each sample including that for the target genes as well as that of the house-keeping gene, *rrsG,* which was used to normalize the data. Fold were calculated as described earlier ([4](#_ENREF_4)):

2^-((C_t_ *_pCV1,i_ -* C_t_ *_rrsG-CV1,i_*) - (C_t_ *_mqsRAC,i_* - C_t_ *_rrsG-mqsRAC,i_*))

where C_t_ *_mqsRAC,i_*___is the cycle number for *rmf, hpf,* or *raiA* mRNA in MG1655*/*pCV1-mqsRAC, C_t_ *_pCV1,i_*___is the cycle number for *rmf, hpf,* or *raiA* mRNA in MG1655*/*pCV1, C_t_ *_rrsG-mqsRAC,i_* is the cycle number for the associated housekeeping gene *rrsG* for *rmf, hpf,* or *raiA* mRNA in MG1655*/pCV1-mqsRAC*, C_t_ *_rrsG-pCV1i_* is cycle number for the associated housekeeping gene *rrsG* for *rmf, hpf,* or *raiA* mRNA in MG1655*/pCV1*, ∆C_T_ is the difference of the target gene cycle number and the housekeeping cycle number, ∆∆C_T_ is the difference of the MG1655*/pCV1-mqsRAC* ∆C_T_ vs. the MG1655*/pCV1* ∆C_T_, and fold indicates the fold change of the relative expression of the target gene in MG1655*/pCV1-mqsRAC* vs. MG1655*/pCV1.*

| **Gene** | ***rrsG*** | | ***rmf*** | | ***hpf*** | | ***raiA*** | |
| --- | --- | --- | --- | --- | --- | --- | --- | --- |
| Strain | MG1655/pCV1 | MG1655*/pCV1-mqsRAC* | MG1655/pCV1 | MG1655*/pCV1-mqsRAC* | MG1655/pCV1 | MG1655*/pCV1-mqsRAC* | MG1655/pCV1 | MG1655*/pCV1-mqsRAC* |
| C_T_ | 4.6 | 4.48 | 16.9 | 17.0 | 15.3 | 15.2 | 12.8 | 14.7 |
|  | ± 0.5 | ± 0.05 | ± 0.6 | ± 0.2 | ± 0.6 | ± 0.1 | ± 0.2 | ± 0.1 |
| ∆C_T_ |  |  | 12.3 | 12.6 | 10.7 | 10.7 | 8.2 | 10.2 |
|  |  |  | ± 0.1 | ± 0.2 | ± 0.2 | ± 0.1 | ± 0.3 | ± 0.2 |
| ∆∆C_T_ |  |  | 0.3 |  | 0.02 |  | 2.0 |  |
|  |  |  | ± 0.1 |  | ± 0.02 |  | ± 0.1 |  |
| Fold |  |  | 1.2 |  | 1.05 |  | 4.0 |  |

**Supplemental Figure S1. Growth of four independent colonies of each strain in LB at 37^o^C.** MG1655, the wild-type host, is indicated in red, MG1655/pCV1, the host with the empty plasmid (negative control), is indicated in orange, and MG1655/pCV1-mqsRAC, the host with the MqsR/MqsA/MqsC tripartite toxin/antitoxin system, is indicated in blue. One average deviation shown.


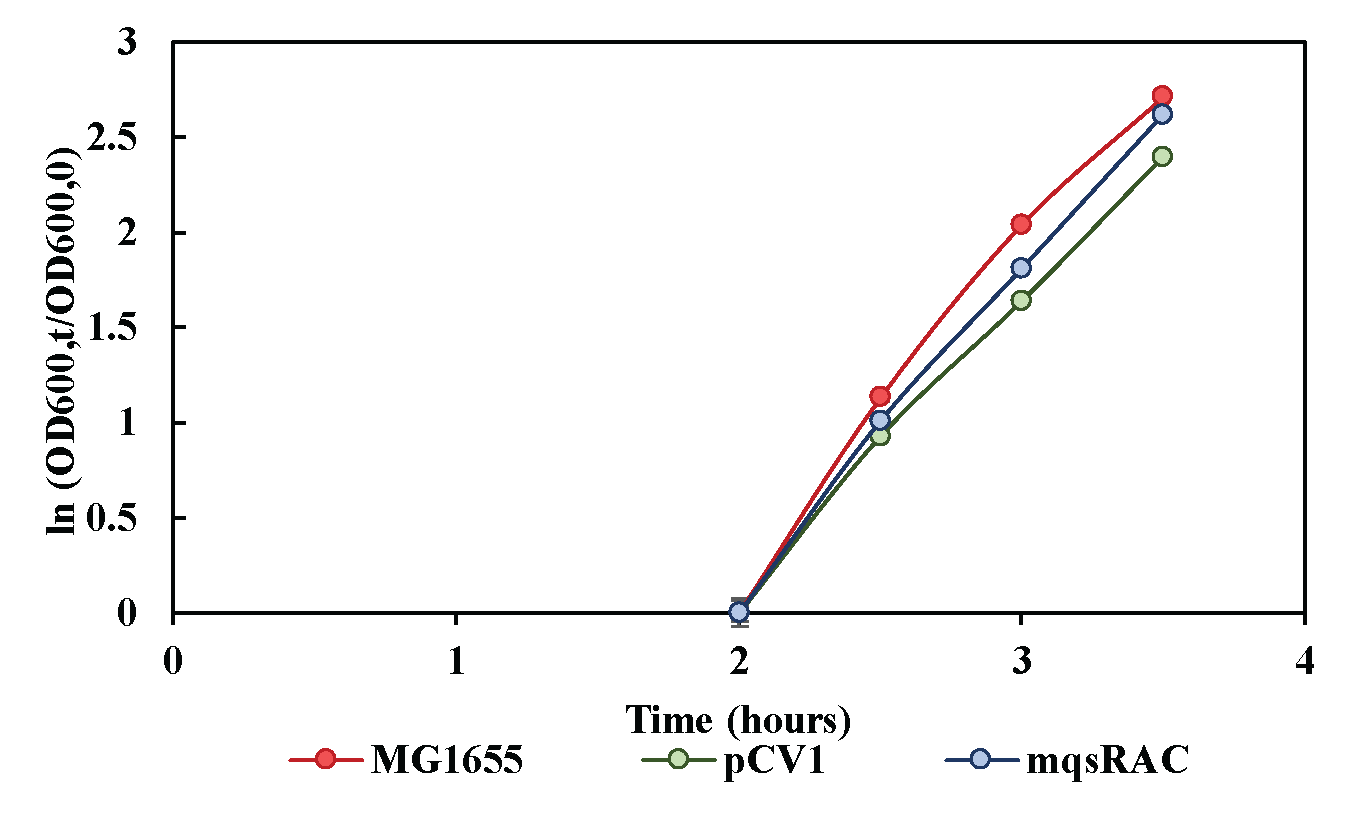


**Supplemental Figure S2. Long-term heterogenous single-cell resuscitation after phage attack.** Representative images (from five independent cultures) of the resuscitation of *E. coli* persister cells with MqsR/MqsA/MqsC (**upper row**) and division of exponential cells (**lower row**) after 0 to 3 h as determined with light microscopy (Zeiss Axio Scope.A1) using LB agarose gel pads. The persister cells were generated by the addition of T2 phage at 0.1 MOI for 1 hour. Cells with the empty plasmid (i.e., no MqsR/MqsA/MqsC) are not shown due to the cellular debris that stems from complete eradication by T2 phage. Black arrows indicate cells with immediate waking (within 30 min), yellow arrows indicate cells with delayed waking (waking between 30 – 180 min), red arrows indicate cells that wake then die (lyse within 3 h), and blue arrows indicate cells that elongate. Black arrows were not added to exponential cultures pictures because all of them divide. Data for percentages from 0 to 1 h are shown in **Table S5**.


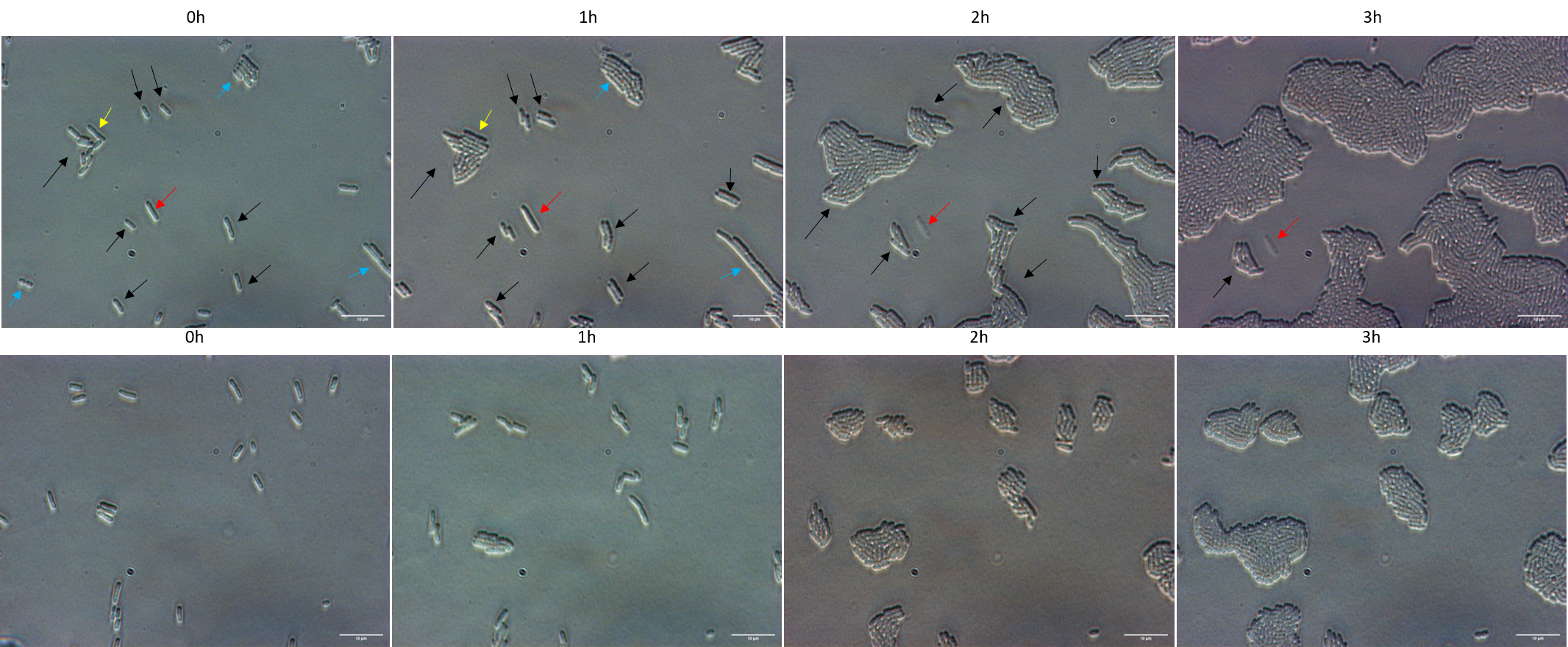


**Supplemental Figure S3. Metabolic activity via flow cytometry.** MG1655/pCV1*-mqsRAC* was grown to a turbidity of 0.5 at 600 nm and T2 phage was added (MOI 0.1) for 1 h, then stained for metabolic activity using the BacLight™ RedoxSensor™ Green Vitality Kit (‘persister’ cells, green) and compared to exponentially-growing cells stained with the same kit (‘Exponential culture’, red). RedoxSensor Green measures the cellular redox state. ‘Unstained’ indicates results with an overnight, unstained sample. ‘%Max’ indicates the relative number of cells (100,000 cells total), and ‘FITC-A’ is indicative of the intensity of the RedoxSensor Green fluorescence. One representative image of two independent cultures shown.


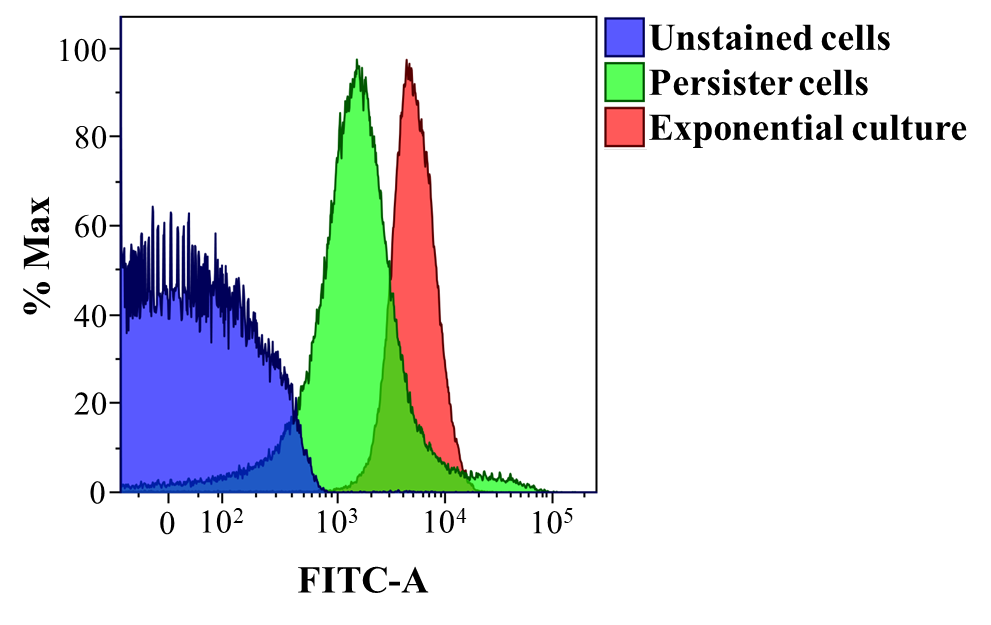


**Supplemental Figure S4. Transmission electron microscopy of *E. coli* cells after T2 phage attack.** MG1655/pCV1-mqsRAC was grown to a turbidity of 0.5 at 600 nm, and T2 phage was added (MOI 0.1) for 1 h. (**A**) exponentially-growing cells prior to phage addition, (**B**) persister cells formed after T2 attack, which are smaller, and spheroid compared to exponentially-growing cells, and (**C**) damaged cells after T2 phage infection. Phage T2 indicated with an arrow and labelled ‘Bacteriophage’.


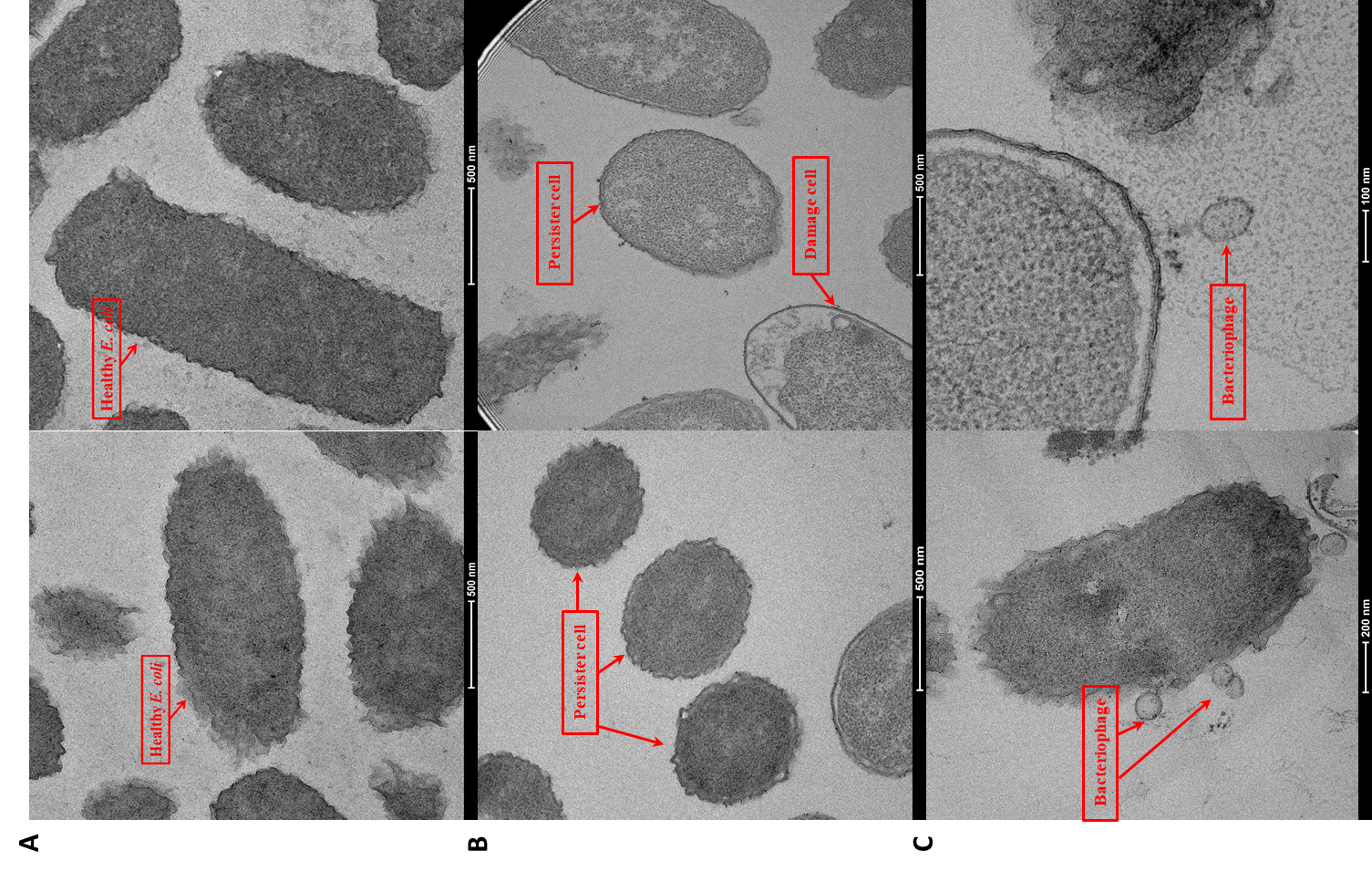


REFERENCES

1. Blattner, F.R., III, G.P., Bloch, C.A., Perna, N.T., Burland, V., Riley, M., Collado-Vides, J., Glasner, J.D., Rode, C.K., Mayhew, G.F. *et al.* (1997) The complete genome sequence of *Escherichia coli* K-12. *Science*, **277**, 1453-1462.

2. Baba, T., Ara, T., Hasegawa, M., Takai, Y., Okumura, Y., Baba, M., Datsenko, K.A., Tomita, M., Wanner, B.L. and Mori, H. (2006) Construction of *Escherichia coli* K-12 in-frame, single-gene knockout mutants: the Keio collection. *Mol Syst Biol*, **2**, 2006 0008.

3. Vassallo, C.N., Doering, C.R., Littlehale, M.L., Teodoro, G.I.C. and Laub, M.T. (2022) A functional selection reveals previously undetected anti-phage defence systems in the *E. coli* pangenome. *Nature Microbiology*, **7**, 1568-1579.

4. Pfaffl, M.W. (2001) A new mathematical model for relative quantification in real-time RT-PCR. *Nucleic Acid Res*, **29**, e45.
